# The hydration of Li^+^ and Mg^2+^ in subnano carbon nanotubes using a multiscale theoretical approach

**DOI:** 10.3389/fchem.2023.1103792

**Published:** 2023-02-02

**Authors:** Ruirui Liu, Zhuanfang Jing, Yifan Shao, Yongquan Zhou, Fayan Zhu, Hongyan Liu

**Affiliations:** ^1^ Key Laboratory of Comprehensive and Highly Efficient Utilization of Salt Lake Resources, Qinghai Institute of Salt Lakes, Chinese Academy of Sciences, Xining, Qinghai, China; ^2^ Key Laboratory of Salt Lake Resources Chemistry of Qinghai Province, Qinghai Institute of Salt Lakes, Chinese Academy of Sciences, Xining, Qinghai, China

**Keywords:** carbon nanotube (CNT), hydration structure, molecular dynamics simulation, separation, multiscale theoretical approach, Li^+^ and Mg^2+^

## Abstract

The separation of brines with high Mg/Li mass ratios is a huge challenge. To provide a theoretical basis for the design of separation materials, the hydration of Li^+^ and Mg^2+^ in confinement using carbon nanotubes (CNTs) as the 1-D nanopore model was investigated using a multiscale theoretical approach. According to the analysis of the first coordination layer of cations, we determined that the coordination shells of two cations exist inside CNTs, while the second coordination shells of the cations are unstable. Moreover, the results of the structure analysis indicate that the hydration layer of Li^+^ is not complete in CNTs with diameters of 0.73, 0.87, and 1.00 nm. However, this does not occur in the 0.60 nm CNT, which is explained by the formation of contact ion pairs (CIP) between Li^+^ and Cl^−^ that go through a unstable solvent-shared ion pair [Li(H_2_O)_4_]^+^, and this research was further extended by 400 ns in the 0.60 nm CNT to address the aforementioned results. However, the hydration layer of Mg^2+^ is complete and not sensitive to the diameter of CNTs using molecular dynamics simulation and an *ab initio* molecular dynamics (AIMD) method. Furthermore, the results of the orientation distribution of Li^+^ and Mg^2+^ indicate that the water molecules around Mg^2+^ are more ordered than water molecules around Li^+^ in the CNTs and are more analogous to the bulk solution. We conclude that it is energetically unfavorable to confine Li^+^ inside the 0.60-nm diameter CNT, while it is favorable for confining Li^+^ inside the other four CNTs and Mg^2+^ in all CNTs, which is driven by the strong electrostatic interaction between cations and Cl^−^. In addition, the interaction between cations and water molecules in the five CNTs was also analyzed from the non-covalent interaction (NCI) perspective by AIMD.

## 1 Introduction

The flux and selective rate of various ions in porous materials is a key issue in the desalination or purification of salt lake brine (Mashl et al., 2003; Qiao et al., 2006; Shao et al., 2007). CNTs are listed as a good prototype for 1-D nanopores for various types of nanopores because they can easily represent the confinement effect of porous materials and the diameters are easy to adjust (Wang et al., 2004; Varshney et al., 2018). The confined fluids inside the nanopore show properties that are different from those of their bulk counterparts (Rasaiah et al., 2008; Mohammed and Gadikota, 2018; Liu et al., 2020), which is caused by anisotropic interactions and geometric constraints (Gelb et al., 1999). For example, water molecules present ultrafast flow in membranes with sub2-nm pores (Holt et al., 2006). Furthermore, a number of new ice phases are not observed in the bulk ice but are found in carbon nanotubes (CNTs) (Koga et al., 2001). These anomalies mainly arise from the structural changes of fluid molecules. Therefore, ion hydration in nanopores may have its own peculiarities.

The ionic hydration structure is important for the selectivity and flux of ions inside nanopores (Di Leo and Maranon, 2005; Carrillo-Tripp et al., 2006; Noskov and Roux, 2007). Many anomalous ionic hydration behaviors are not observed in their bulk solution but are found under confinement by molecular dynamics (MD) simulation (Yan et al., 2015; Foroutan et al., 2017). For instance, Shao et al. showed that confinement changes the temperature dependence of water molecule’s structure order in the first coordination shell of K^+^ through an MD simulation (Shao et al., 2008). In a (10, 10) CNT, an increase in temperature results in an order enhancement instead of a decrease, which was observed in the bulk solution (Shao et al., 2008). Moreover, the pore size effect on the hydration structures of cations was also evaluated (Shao et al., 2009), and results indicate that a minor change in the CNT diameter can cause an obvious structural change in the water molecules around the cations. Zhou et al. found an abnormal ion association between Ca^2+^ and Cl^−^ in 1.09 nm–1.22 nm CNTs (Wang et al., 2022). In this case, to better explore the transfer and behavior of confined fluids, their structure should be studied in detail (Pal et al., 2021). MD simulation is a versatile method that can be used to study the structure of confined fluids at the atomic level (Marti et al., 2010; Salles et al., 2011; Horstmann et al., 2022).

Membrane technology comprises various selective membranes with unique structures for separating Li^+^ from Mg^2+^ in brines and seawater, which has previously been confirmed from a theoretical point of view (Yang et al., 2013; Azamat et al., 2016). This provides an effective approach for lithium extraction from aqueous solutions containing multiple coexisting ions (Na^+^, K^+^, Ca^2+^, Mg^2+^, etc.) (Nie et al., 2017; Xu et al., 2019). However, there has been slow development in industrial applications as a result of the complexity of actual brines and the seawater system, especially those with high Mg/Li mass ratios (Zhang et al., 2019; Ying et al., 2020). Therefore, the hydration of Li^+^ and Mg^2+^ in confined spaces may provide a theoretical basis for the design of separation materials.

In this study, we performed a multiscale theoretical approach to examine the hydration of two different cations (Li^+^ and Mg^2+^) inside CNTs with diameters of approximately 0.60, 0.73, 0.87, 1.00, and 1.28 nm. Detailed structural information was analyzed using different theoretical methods. We were also interested in comparing the shell order variations of Li^+^ and Mg^2+^ inside CNTs and the related impacts on the hydration of these two cations. Then, the pairwise interaction energies, interaction energies, and binding energies among cations, CNTs, and water molecules were analyzed by quantum chemistry and molecular dynamics simulations. Finally, the weak interaction between the CNTs and water molecules was analyzed in detail by the non-covalent interaction (NCI) method.

## 2 Simulation model and methods

The hydration of Li^+^ and Mg^2+^ inside five single-walled infinite armchair CNTs with diameters of 0.60, 0.73, 0.87, 1.00, and 1.28 nm was performed by MD simulation at 298 K. CNTs were built through the molecular visualization program (VMD). It is easier to form an ion pair in high-concentration solutions, which may make observing ion hydration a challenge. Therefore, the lengths of CNTs with different diameters are different, which ensures that every CNT contains a considerable number of water molecules and a low ionic concentration inside the CNTs. The corresponding lengths of CNTs with diameters of 0.60, 0.73, 0.86, 1.00, and 1.28 nm are 24.6, 27.1, 20.0, 14.2, and 8.4 nm, respectively.

The simulation systems are built with the following process. First, certain water molecules are randomly placed inside the CNTs. After energy minimization, two water molecules are randomly chosen for replacement by a cation and an anion. As shown in Supplementary Figure S1, the initial distances of the cation and anion are 3.8, 4.0, and 4.2 nm in three parallel systems as followed by Shao et al. (2008). The details of all simulation cases are listed in Supplementary Table S1. In every case, three individual MD simulations with different initial positions and velocities for water molecules and ions were carried out to ensure the reliability of the results.

All molecular dynamics simulations were carried out using Gromacs 2019.4 software (Kutzner et al., 2015; Pall et al., 2020). The OPLS-AA force field was used to describe LiCl, MgCl_2_, and CNT systems (Jorgensen et al., 1996). Supplementary Table S2 lists the L-J parameters and the partial charges used in this study (Shao et al., 2008). The potential energy of intermolecular interactions is described as a combination of the L-J 12-6 and coulombic potential

$$U\left(r_{ij}\right)=4\varepsilon_{ij}\left[{\left(\frac{\sigma_{ij}}{r_{ij}}\right)}^{12}-{\left(\frac{\sigma_{ij}}{r_{ij}}\right)}^{6}\right]+\frac{q_{i}q_{j}}{r_{ij}},$$ (1)

where *r*_*ij*_ is the distance between atom *i* and atom *j* and *q*_*i*_ is the partial charge that was assigned to atom *i*. LiCl, MgCl_2_, and CNTs are solvated in cubic water boxes where SPC/E water molecules are used with periodic boundary conditions (Berendsen et al., 1984). The size of the boxes and the number of water molecules are listed in Supplementary Table S3. The cut-off of the van der Waals (vdWs) interaction and the grid spacing of the long-range electrostatic interaction were set to 1.0 and 1.5 nm, respectively. The steep method was used to execute energy minimization. The SHAKE algorithm was used to restrict all bonds including hydrogen atoms. To keep the diameter of the carbon nanotube steady, the coordinates of the CNTs were frozen during the MD run. A production simulation phase was conducted in the NVT ensemble using the Berendsen thermostat with a coupling coefficient of τT = 0.1 ps. For each configuration, 100 ns of data-production simulations were conducted. A time step of 2.0 fs was used, and data were collected every 20 ps. The molecular visualization program VMD was used to obtain snapshots of the key configuration (Humphrey et al., 1996).

## 3 Results and discussion

### 3.1 Hydration structure of Li^+^ and Mg^2+^ inside CNTs

**FIGURE 1:**
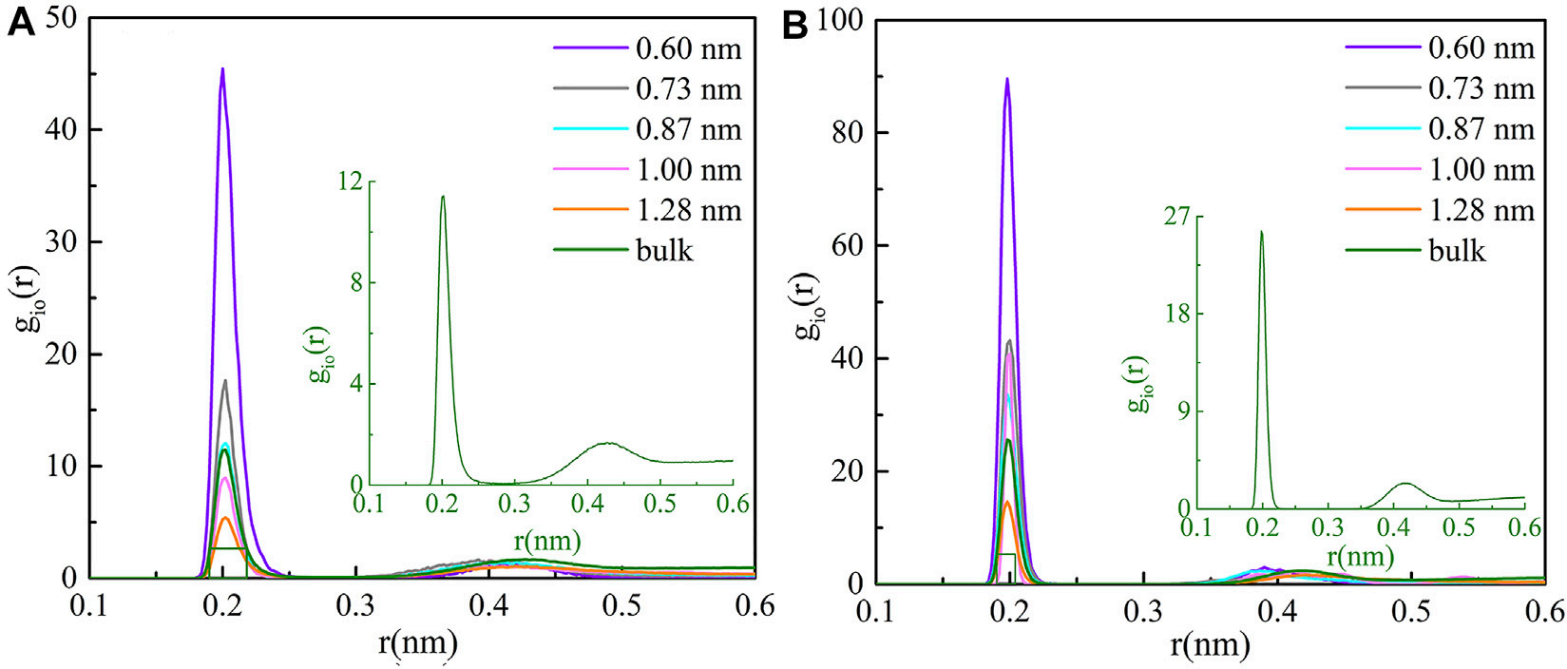
Ion RDFs for **(A)** Li^+^ and **(B)** Mg^2+^ inside various CNTs and the bulk solution.

As shown in Figure 1, the radial distribution functions (RDFs) of cations and oxygen atoms inside the five CNTs and bulk solution are calculated with the trajectory. We found that the radial distribution of O atoms around the two cations inside CNTs shows a maximum value and a minimum value, and they are both smaller than the radius of CNTs, which indicates that the coordination shells of two cations exist inside CNTs. The second peaks of RDFs are obviously weak in all cases, indicating that the second coordination shells of the cations may be unstable in all CNTs. At the same time, this phenomenon further confirms the rationality of all models.

**FIGURE 2:**
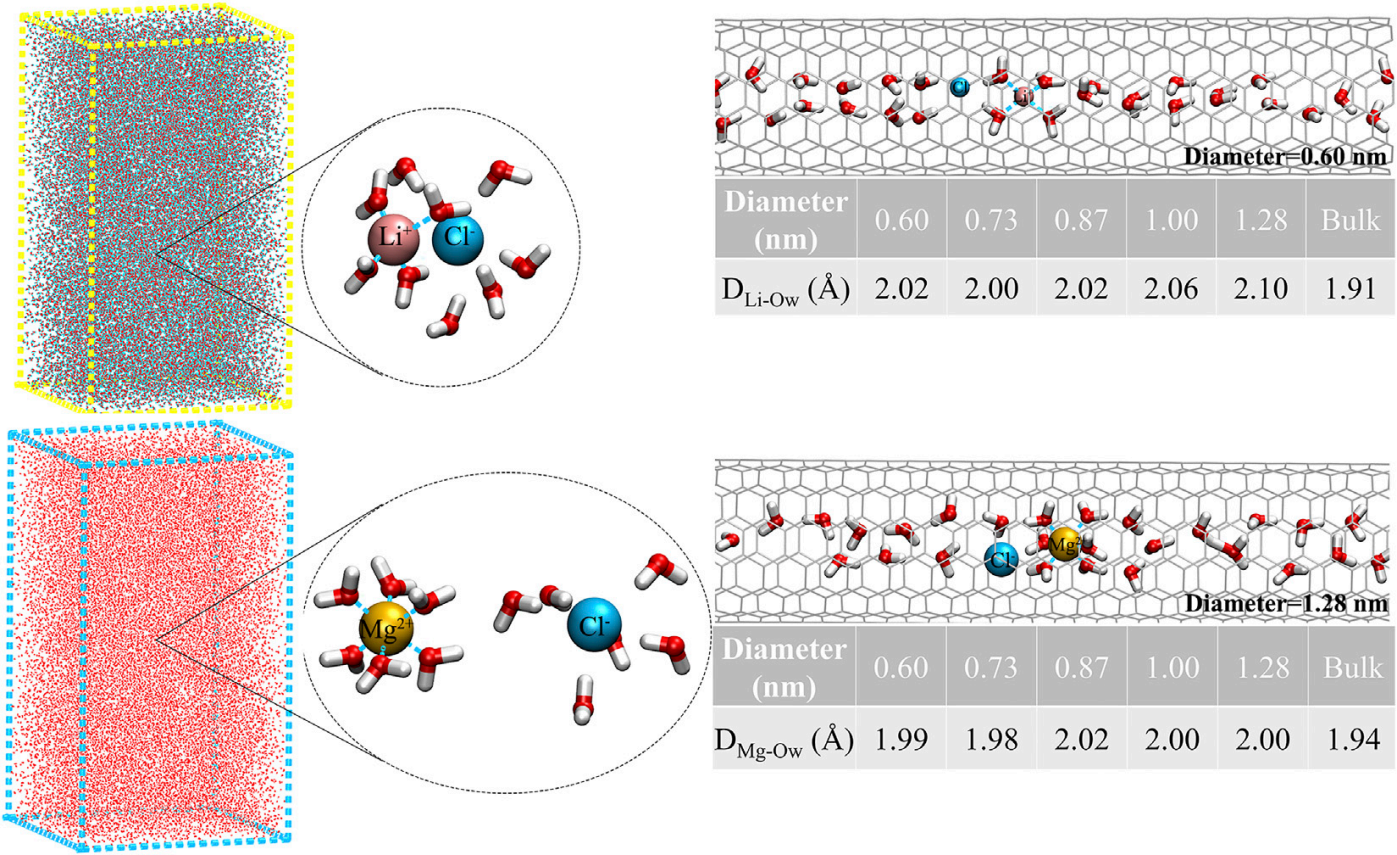
Hydration structure of ions and the average bond lengths of cations and water molecules, Li^+^ and Cl^−^ in the bulk solution, and the five CNTs.

To better study the detailed information of the coordination shell, the distances of the first maximum (*r*_*max*_) and minimum (*r*_*min*_) of cations and oxygen atoms inside the five CNTs and bulk solution are listed in Table 1. We observed that the *r*_*max*_ and *r*_*min*_ for Li^+^ were 2.0 and 2.6 Å inside CNTs, respectively, agreeing well with the corresponding bulk values observed in this work and the simulation results of others (Rasaiah et al., 2000; Zhou et al., 2002). The *r*_*max*_ and *r*_*min*_ values of 2.0 and 2.3 Å, respectively, were observed for confined Mg^2+^ inside all CNTs, which was similar to the findings of Pettitt and Rossky (1986). Table 1 also lists the coordination numbers (Nc) of water molecules around Li^+^ and Mg^2+^ in CNTs and the bulk solution. We found that the Nc of water molecules around Mg^2+^ remains 6.0 in all five CNTs and is consistent with its Nc in the bulk solution and the simulation results of other studies (Pettitt and Rossky, 1986; Rasaiah et al., 2000), which suggests that the Nc of water molecules around Mg^2+^ is not sensitive to the diameters of CNTs. Compared with the bulk solution, the Nc of water molecules around Li^+^ inside the CNTs with diameters of 1.28, 1.00, 0.87, and 0.73 nm has a different reduction, which is 3.7 inside 1.28 nm CNTs and 0.4 smaller than that of the bulk solution, while the other value is close to 3.0 in CNTs with diameters of 1.00, 0.87, and 0.73 nm, which is 1.1 smaller than the bulk counterpart. However, a different phenomenon occurs in the CNT with a diameter of 0.60 nm in which a complete first coordination shell is observed for Li^+^ compared to the 0.73, 0.87, and 1.00 nm CNTs, which indicates that the confinement effect of 0.60 nm CNT on Li^+^ is more obvious. In summary, the first coordination shell of Li^+^ is no longer complete in CNTs with diameters of 0.73, 0.87, and 1.00 nm, yet this case does not occur in the five CNTs for Mg^2+^. Furthermore, to clearly observe the first coordination shells of Li^+^ and Mg^2+^ in the five CNTs, the hydration structure of cations and the average bond lengths of cations and the O atoms of water molecules (Ow) in the bulk solution and CNTs are presented in Figure 2. The first coordination layer of Li^+^, constituted by four water molecules in the 0.60 nm CNT and the bulk solution and containing three water molecules and one Cl^−^ in the 0.73, 0.87, and 1.00 nm CNTs, was observed. These observations indicate that the 4-coordinated Li^+^ is more stable. Moreover, we determined that the average bond length between Li^+^ and Ow is 2.02 Å inside the CNT with a diameter 0.60 nm, and it is significantly longer by 0.11 Å than that in the bulk solution. This foundation indicates that the hydration layer of Li^+^ is looser in the 0.60 nm CNT. While the distances of Li^+^-Ow were similar in the 0.73, 0.87, and 1.00 nm CNTs, Cl^−^ may form the first coordination layer of Li^+^. Similar to the bulk solution, Li^+^ has four coordination with CNTs, and the reason for the difference in Nc is that Cl^−^ participates in the formation of the first coordination layer. We also observed that the bond length between Mg^2+^ and Ow in CNTs is nearly 3.0% larger than that in the bulk solution, which suggests that the first coordination layer of Mg^2+^ in the five CNTs is also looser than that in the bulk solution. Unlike Li^+^, Cl^−^ does not participate in the formation of the first coordination layer of Mg^2+^ in the bulk solution and CNTs.

**TABLE 1:** Position of the first maximum (r_max_) and first minimum (r_min_) of the ion-oxygen RDFs for Li^+^ and Mg^2+^ and the coordination number (Nc) of their first coordination shells.

Diameter (nm)	Li^+^			Mg^2+^		
	r_max_(Å)	r_min_(Å)	N_c_	r_max_(Å)	r_min_(Å)	N_c_
0.60	2.0	2.6	4.4	2.0	2.3	6.0
0.73	2.0	2.4	3.0	2.0	2.3	6.0
0.87	2.0	2.4	3.0	2.0	2.3	6.0
1.00	2.0	2.5	3.2	2.0	2.2	6.0
1.28	2.0	2.6	3.7	2.0	2.3	6.0
bulk	2.0	2.7	4.1	2.0	2.3	6.0

To determine why Cl^−^ does not participate in the formation of the first coordination layer of Li^+^ in the 0.6 nm CNT, the simulation time of this system was extended by 400 ns under the same conditions. The distance between Li^+^ and Cl^−^ in the last 400 ns and the corresponding structure are shown in Supplementary Figure S2. Unexpectedly, the distance between Li^+^ and Cl^−^ was reduced to 2.29 Å at 341 ns, after which three water molecules occur around Li^+^, and Cl^−^ also participates in the formation of the first coordination layer, which is the same as in the 0.73, 0.87, and 1.00 nm CNTs. This phenomenon shows that the confinement effect of smaller CNTs makes the formation of contact ion pairs (CIPs) more difficult. Then, *ab initio* molecular dynamics (AIMD) simulations were performed using the CP2K program (Kuhne et al., 2020) to confirm the aforementioned results, and the simulation details are described in the Supplementary Simulation Methods Section. CNTs with diameters of 0.60, 0.73, and 1.28 nm were selected and studied. As shown in Supplementary Figure S3, the distance between Li^+^ and Cl^−^ is larger at 2.83–4.55 ps in the 0.60 nm CNT, which illustrates that Cl^−^ is insufficient to participate in the formation of the first coordination layer of Li^+^, and the distance decreases to 2.32 Å, manifesting the CIP of Li^+^ and Cl^−^ forms over the last time. These observations indicate that the formation of CIP between Li^+^ and Cl^−^ requires an intermediate [Li(H_2_O)_4_]^+^ in the 0.60 nm CNT. The distance between Li^+^ and Cl^−^ is directly reduced to 2.18 and 2.22 Å in a very short time in CNTs with diameters of 0.73 and 1.28 nm, and the coordination form of Li^+^ is [Li(H_2_O)_3_]^+^Cl^−^. From these results, we conclude that the required time for Cl^−^ to participate in the formation of the first coordination layer of Li^+^ is as follows: CNT (7, 7) > CNT (11, 11) > CNT (8,8), which is consistent with the previously presented trend for the Nc of water molecules around Li^+^ and demonstrates the consistency of MD and AIMD results.

**FIGURE 3:**
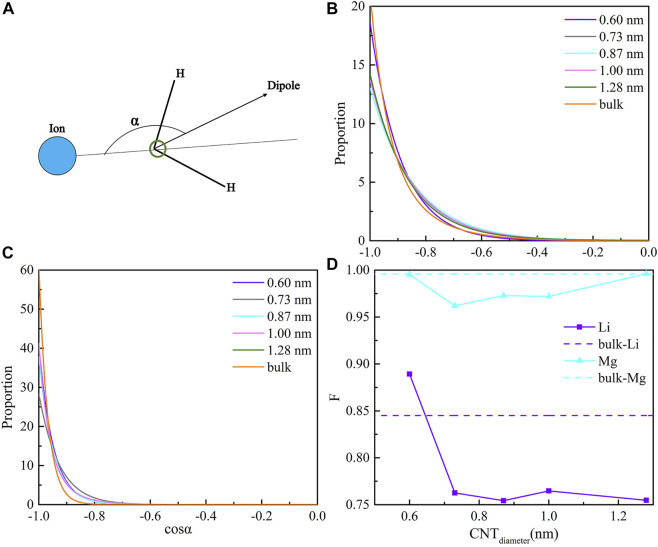
Coordination shell order. **(A)** definition of angle α; **(B)** distributions of cosα for Li^+^ in CNT and the bulk solution; **(C)** distributions of cosα for Mg^2+^ in CNTs and the bulk solution; and **(D)** hydration factor F for Li^+^ and Mg^2+^ inside CNTs as a function of diameter.

Based on the electrostatic interaction between ions and water molecules, the water molecules around the ions show a certain orientation distribution. An orientation distribution function was proposed to characterize the orientation structure of water molecules around an ion by Zhou et al. (2002). It is defined as the distribution probability of the orientation angle between ions and water molecules. The orientation angle is presented in Figure 3A. The orientation distribution function of the water molecules around Li^+^ and Mg^2+^ inside the CNTs and bulk solution are shown in Figures 3B,C. The orientation of water molecules around Li^+^ is strongest in the bulk solution and CNTs with a diameter of 0.60 nm, yet the Li^+^ inside the CNTs with diameters of 1.28, 0.73, 1.00, and 0.87 nm takes third, fourth, fifth, and sixth place, respectively. For Mg^2+^, we observe that the maximum values of the distribution probability of the orientation angle in the CNTs with diameters of 0.60, 0.73, 0.87, 1.00, and 1.28 nm are 40.51, 28.27, 37.81, 41.23, and 60.73, respectively, and that in the bulk solution is 60.73, which are significantly larger than those of Li^+^ inside all CNTs. This indicates that the water molecules around Mg^2+^ are more ordered than those around Li^+^ under the confinement of CNTs. Moreover, their cosine values of the orientation angle present a maximum at −1, which shows the water molecules around ions approaching Li^+^ and Mg^2+^ with the O atoms and the H atoms away from the ions. The results show that the order degree of the water shell around the ions is proportional to the Nc of the water molecules around cations.

To further investigate the variation in shell order, we applied the hydration factor (F) parameter proposed by Zhou et al. (2002) and defined in Eq 2:

$$F=\frac{N_{-1<\cos\alpha <-0.72}^{firstshell}}{N_{all}^{firstshell}}.$$ (2)

F of Li^+^ (F_Li_) and Mg^2+^ (F_Mg_) inside the five CNTs and the bulk solution as a function of CNT diameter are shown in Figure 3D. We can observe two types of diameter dependences for F_Li_. Inside the four CNTs with diameters of 0.73, 0.87, 1.00, and 1.28 nm, the F_Li_ values are 0.76, 0.75, 0.76, and 0.75, respectively, which are nearly the same compared to each other and all smaller than the bulk one by approximately 10.59% and 11.76%, which indicates that the shell order of Li^+^ is nearly identical inside these four CNTs. Both of these cases imply that the hydration of Li^+^ is nearly identical in these four CNTs, which is weaker than that of the bulk solution. However, we found that there is a considerable increase in F_Li_ occurring in the narrowest CNT with a diameter of 0.60 nm in Figure 3D, and it is nearly 4.7% larger than that in the bulk one. The Nc of Li^+^ inside this CNT is also nearly 4.7% compared with that of the bulk solution, which indicates that the hydration of Li^+^ inside this CNT may be similar to that in the bulk solution within 100 ns. In addition, we determined that F_Mg_ is nearly 0%, 0.3%, 0.2%, 0.2%, and 0% inside the five CNTs with diameters of 0.60, 0.73, 0.87, 1.00, and 1.28 nm, respectively, compared to that in the bulk solution, which implies that the hydration of Mg^2+^ inside all CNTs may be more similar to that in the bulk solution and is not sensitive to the diameter of the CNTs.

### 3.2 The interaction details of ions inside CNTs

**FIGURE 4:**
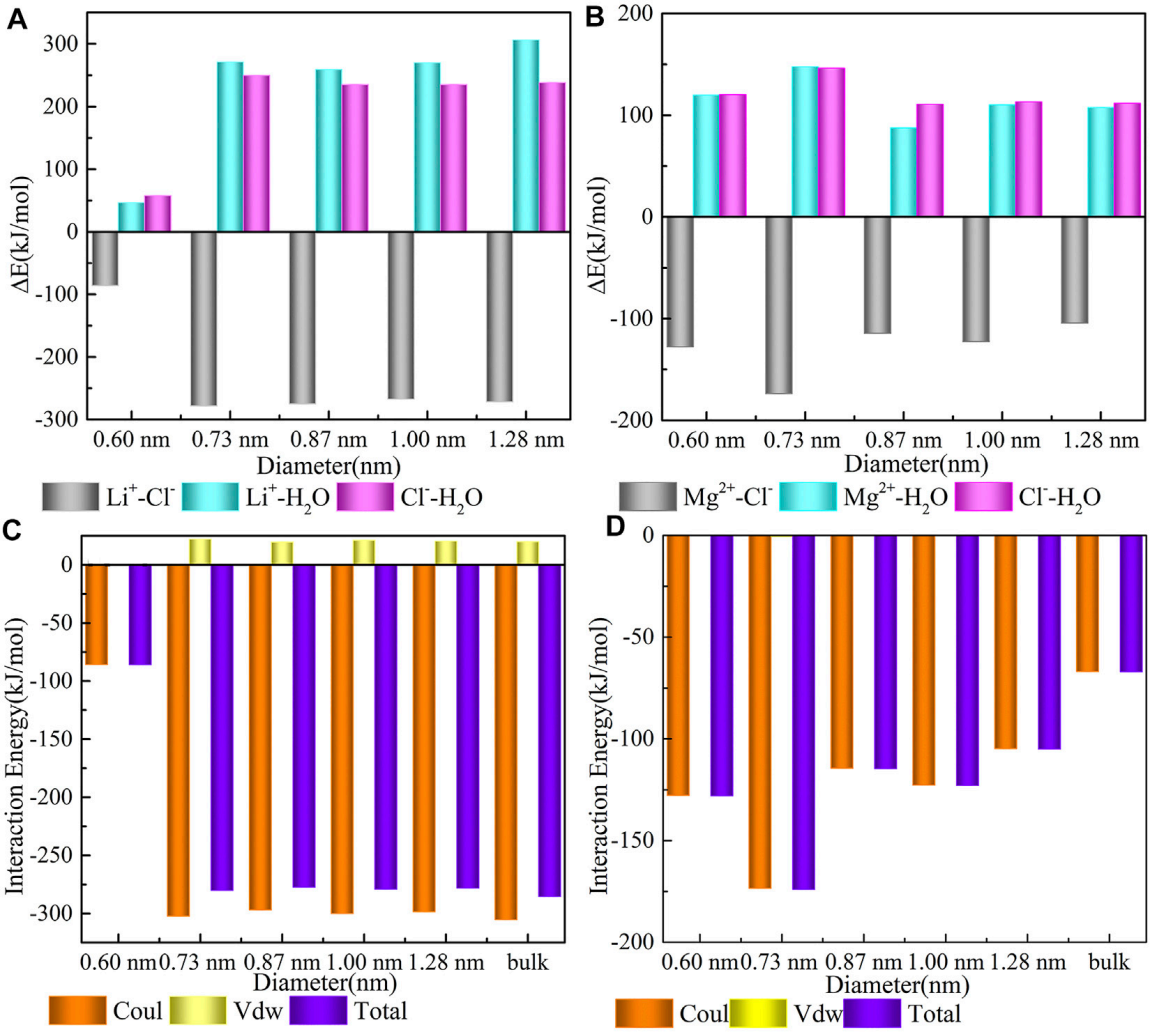
Energy details. **(A,B)** pairwise interaction energies of ions-CNT, cation–water, and anion-water; **(C)** the interaction between Li^+^ and Cl^−^; and **(D)** the interaction between Mg^2+^ and Cl^−^.

As shown in Figure 4, three pairwise interacting energies are divided. The binding of Li^+^ and Cl^−^ released approximately 86.00, 278.18, 274.91, 267.47, and 271.48 kJ/mol energy inside the five CNTs with diameters of 0.60, 0.73, 0.87, 1.00, and 1.28 nm, respectively. Meanwhile, at the interface between Li^+^ and Cl^−^, parts of the water were squeezed out, causing dehydration of Li^+^ and Cl^−^ (Li^+^−water term 46.45, 271.26, 259.18, 269.92, and 306.11 kJ/mol; Cl^−^−water term 57.53, 249.47, 234.85, 234.77, and 238.11 kJ/mol), which were partly compensated by the energy release due to Li^+^ and Cl^-^ interaction. From these data, we know that the interaction energy between Li^+^ and Cl^−^ inside the 0.60 nm CNT shows a maximum value. This observation indicates that the binding of Li^+^ and Cl^−^ in CNTs with a diameter of 0.60 nm is the most difficult, and the difficulty of dehydration of Li^+^ and Cl^−^ in CNTs with other sizes is nearly the same. Additionally, except for the 0.60 nm CNT case, the energy absorbed by the combination of Li^+^ and water molecules is slightly higher than that of the combination of Cl^−^ and water molecules in the CNTs with diameters of 0.73, 0.87, 1.00, and 1.28 nm, which indicates that Li^+^ coordinates with three water molecules [Li(H_2_O)_3_]^+^Cl^−^ in these four CNTs and is more stable than that in the 0.60 nm CNT. The same feature was observed for the confined Mg^2+^, and compared with the Li^+^ system, the required energy for dehydration between Mg^2+^ and Cl^−^ is much lower. This consistency indicates that the dehydration between Mg^2+^ and Cl^−^ is more difficult than that of Li^+^ and its water shell is harder inside the five CNTs. The released energies between Mg^2+^ and Cl^−^ in all CNTs are comparable, which suggests that the binding of Mg^2+^ and Cl^−^ is more stable in CNTs.

As shown in Figures 4C and D, the interaction energies between cations and Cl^−^ are computed during the last 50 ns simulation and includes the following: the electrostatic interaction, vdWs interaction, and total interaction between cations and Cl^−^. We determined that an average driving force of the binding process results from the strong electrostatic interaction between cations and Cl^−^, while the contribution of vdWs is nearly zero. This is caused by the strong electrostatic attraction between positive and negative ions. Consistent with the trend presented in Figure 4B, the interaction energy between Mg^2+^ and Cl^−^ in Figure 4D is comparable in all CNTs. However, the interaction energy between Li^+^ and Cl^−^ in the CNT with a diameter of 0.60 nm shows a different trend from that in the other CNTs in Figure 4C, which is caused by an obvious confined effect on Li^+^ in the 0.60 nm CNT. From an energy perspective, these observations suggest that the hydration of Li^+^ in CNTs with a diameter around 0.60 nm seems to be identical to that observed in the other CNTs.

**FIGURE 5:**
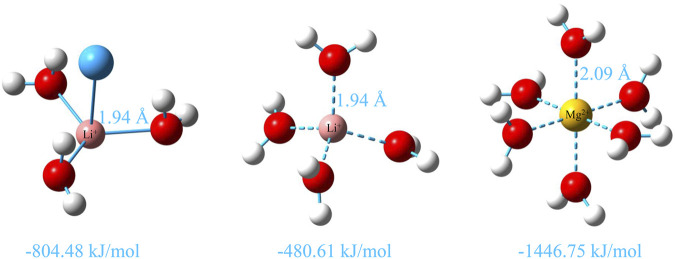
Hydration structures of Li^+^ and Mg^2+^ at the B3LYP/def2-TZVP level (Weigend and Ahlrichs, 2005) and corresponding counterpoise corrected binding energy of Li^+^ and Mg^2+^ with the O atom in water molecules calculated at the PWPB95/def2-QZVPP level (Weigend and Ahlrichs, 2005; Zheng et al., 2011) by ORCA 5.0.3 software (Neese et al., 2020; Neese, 2022), and the D3(BJ) correction and diffuse function are added to all calculations (Grimme et al., 2010; Grimme et al., 2011).

To further explain the coordination situation of cations, the binding energies of Li^+^ and Mg^2+^ with the coordination molecules or ions are calculated by the quantum chemistry method in Figure 5. We determined that the binding energy of 3-coordinated Li^+^ with water molecules is more negative than that of 4-coordinated Li^+^ by −323.77 kJ/mol, which suggests that it is more stable than that of 4-coordinated Li^+^. This is consistent with the analysis results of the previous interaction energy between Li^+^ and Cl^−^. Moreover, the binding energy of 6-coordinated Mg^2+^ with water molecules is −1,446.75 kJ/mol and is the most stable combination mode in which the arrangement of the six water molecules around Mg^2+^ is relatively tight. This also indicates that the 6-coordinated water shell of Mg^2+^ is harder, and the interaction between Mg^2+^ and water molecules is easier than that of Li^+^. Therefore, the interaction between Mg^2+^ and Cl^−^ is weaker than that between Li^+^ and Cl^−^, which is consistent with the description in Figures 4C and D.

**FIGURE 6:**
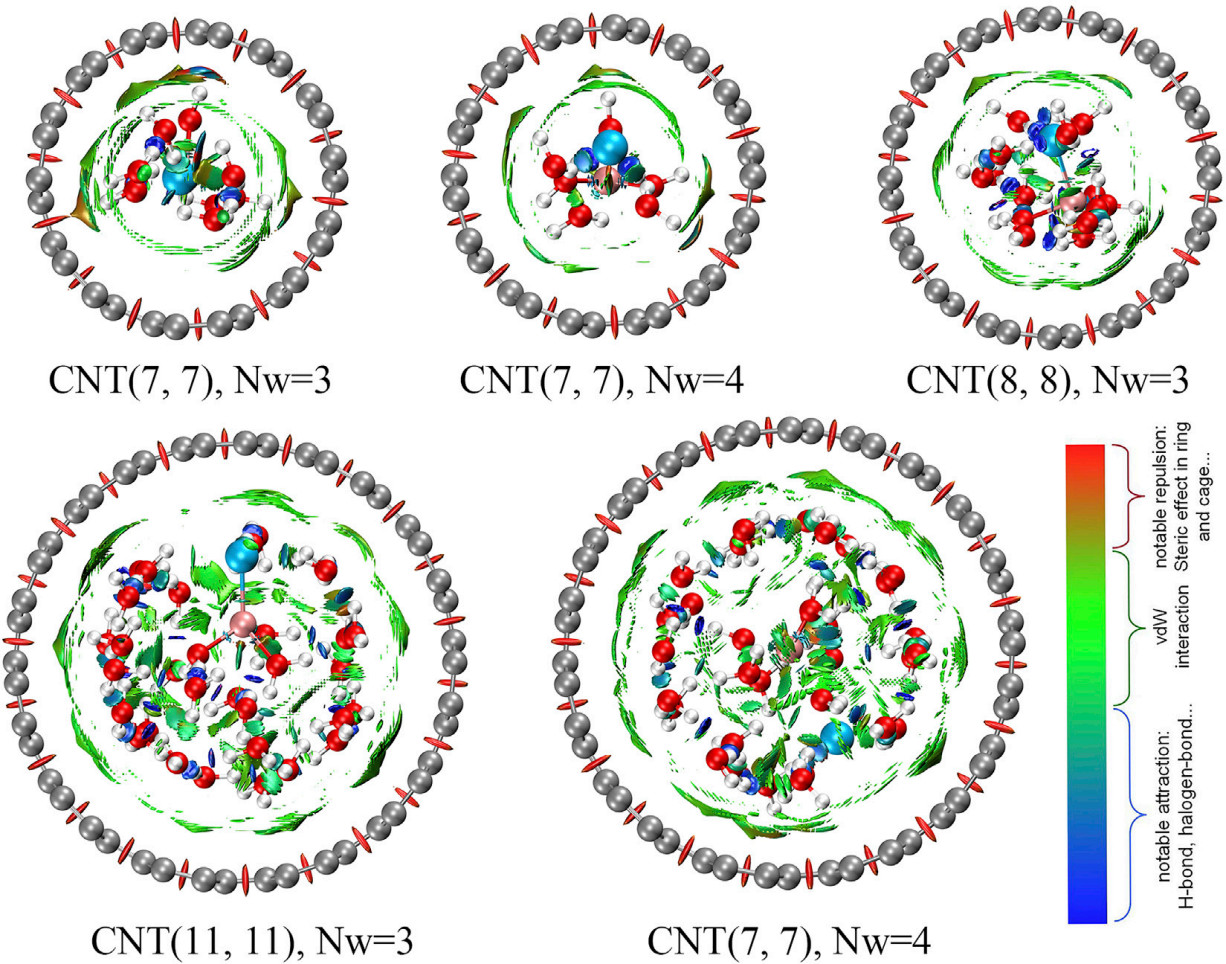
Interaction picture among ions obtained by the non-covalent interaction (NCI) method.

The weak interaction between CNTs and water molecules is shown in Figure 6 to illustrate the differences in the water shell inside the CNTs with diameters of 0.60, 0.73, and 1.28 nm and the different Nc of water molecules around Li^+^ (the details are described in the Supplementary Simulation Methods Section). The color-coding scheme is as follows: blue for attractive, red for repulsive, and green for intermediate interactions, which includes electrostatic and van der Waals interactions. First, the red patch is evenly distributed throughout the CNTs, which indicates that the CNT model is reasonable in this research. We determined that the presence of green patches between the CNTs and water molecules is widespread. In complexes with LiCl and H_2_O in addition to the green patches, blue streaks are also observed, which are caused by hydrogen bonds among water molecules or halogen bonds between Cl^−^ and water molecules. For the LiCl solution inside the 0.60 nm CNT, we observed a smaller area of splattered green patches between the CNT and water molecules (Nw = 4) than in the case of the coordination of three water molecules, which suggests that the interaction between the CNT and water molecules in the [Li(H_2_O)_4_]^+^ form is weaker, and Cl^−^ has difficulty inserting into the first coordination layer of Li^+^ in the 0.60 nm CNT. When Cl^−^ participates in the first coordination layer of Li^+^, the area of green patches between CNTs and water molecules is larger and the interaction is stronger. The distribution of green patches between CNTs and water molecules in the 0.73 nm CNT is wider than that of the 0.60 nm CNT, which indicates that the interaction among water molecules around Li^+^ is weaker, and water molecules are more likely to be displaced by ions so that the CIP of Li^+^ and Cl^−^ is more easily formed in the 0.73 nm CNT. The same cases are analyzed in the 1.28 nm CNT as the 0.60 nm CNT, and the distribution of green patches between CNT and water molecules reaches its maximum when the CIP of Li^+^ and Cl^−^ is formed. However, due to the large number of water molecules contained in the 1.28 nm CNT, the hydrogen bond network between the water molecules is larger and the water molecules are more ordered, which results in insufficient stability in the formation of CIP in the CNT and is the same as the MD results.

## 4 Conclusion

A multiscale theoretical approach was used to study the hydration of Li^+^ and Mg^2+^ confined in CNTs with diameters of 0.60, 0.73, 0.87, 1.00, and 1.28 nm. The results show that the confinement effect of 0.60 nm CNTs on Li^+^ is more obvious, and it is difficult for Cl^−^ to participate in the formation of the first coordination shell of Li^+^, which is caused by the formation of CIP between Li^+^ and Cl^−^ and processing through intermediate [Li(H_2_O)_4_]^+^. However, the formation of CIP between Li^+^ and Cl^−^ is easier in the other four CNTs, and the coordination form of Li^+^ is [Li(H_2_O)_3_]^+^Cl^−^, which is very stable. For Mg^2+^, the water molecules around Mg^2+^ are more ordered and its hydration shell is harder, which is similar to that of the bulk solution. In addition, the execution of AIMD not only confirms some of the previous conclusions, but also explains why Li^+^ is finally coordinated with three waters and one Cl^−^. Finally, due to the weak interaction between CNTs and water molecules, the interaction between CNTs and water molecules in the [Li(H_2_O)_4_]^+^ form is weaker. Additionally, Cl^−^ has difficulty inserting into the first coordination layer of Li^+^ in the 0.60 nm CNT and the water molecules are more ordered, resulting in the formation of CIP in the CNT that is unstable in the 1.28 nm CNT and supports the above results. This work helps elucidate the chemical processes under confined conditions and provides a theoretical reference for the design of Li^+^ and Mg^2+^ separation materials for separating brines and seawater systems with high Mg/Li mass ratios and a size effect.

## Data Availability

The original contributions presented in the study are included in the article/Supplementary Material, and further inquiries can be directed to the corresponding author.
