# Supplementary material for: The hydration of Li+ and Mg2+ in subnano carbon nanotubes using a multiscale theoretical approach
Source: Front Chem. 2023 Feb 2;11:1103792. doi: 10.3389/fchem.2023.1103792 (PMC9932927; doi:10.3389/fchem.2023.1103792)
Supplement: Supplementary file 1 [file DataSheet1.docx]

Supplementary Material

# Supplementary Figures and Tables

## Supplementary Figures

**
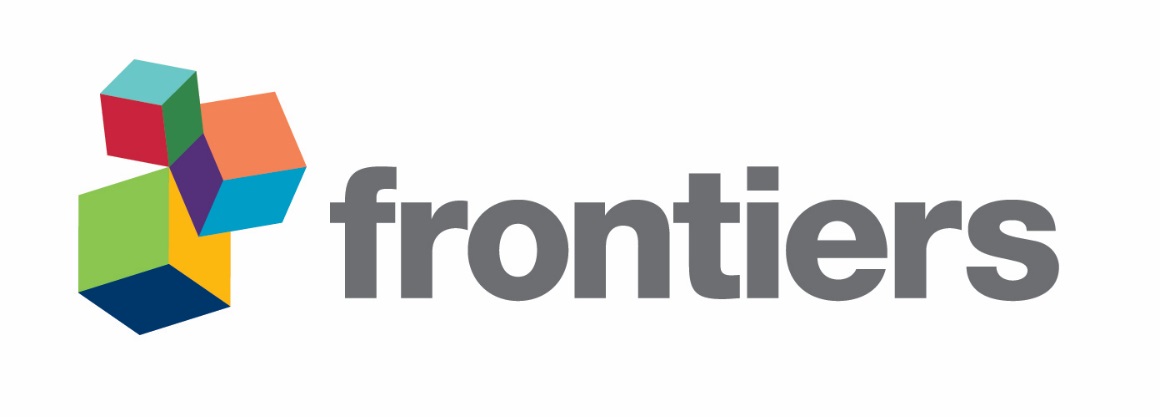
**


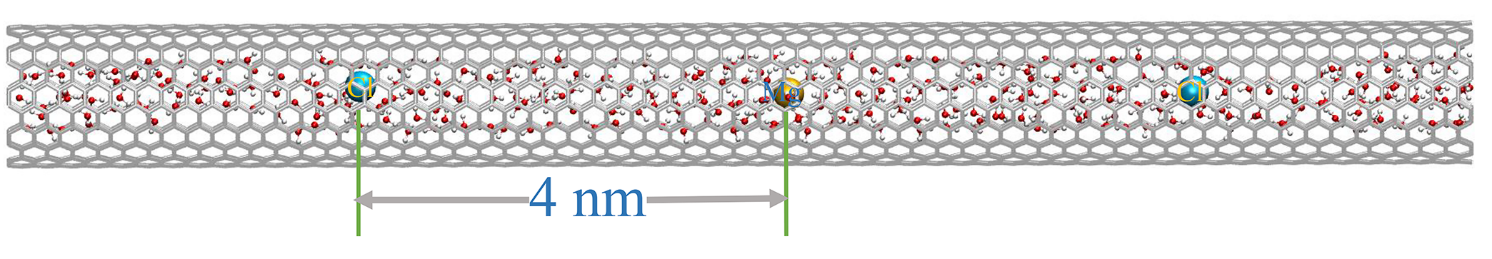


**Supplementary Figure 1.** Schematic illustration of aqueous MgCl_2_ solution-CNT (10, 10) simulation system.

**
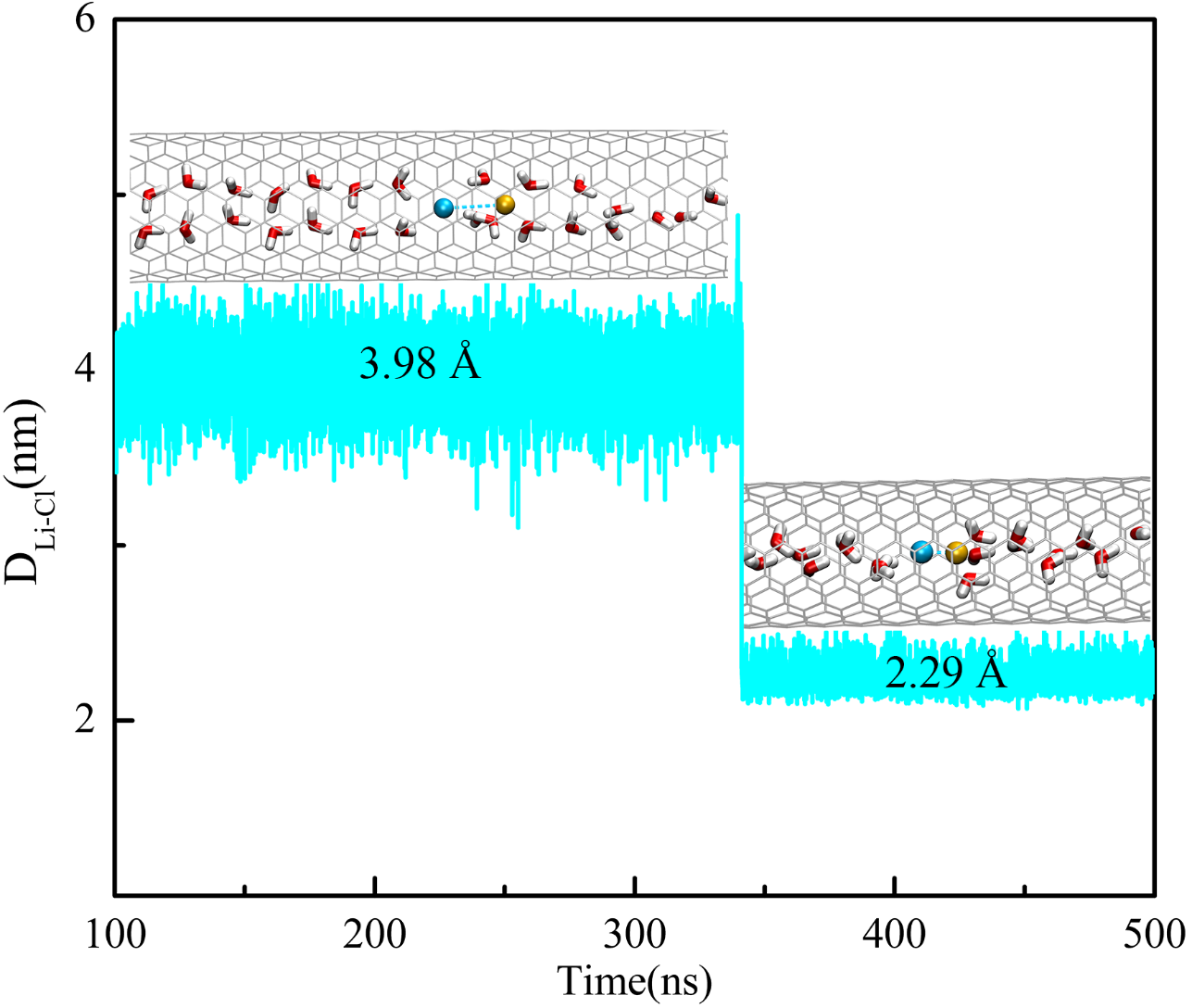
**

**Supplementary Figure 2.** The change of distance between Li^+^ and Cl^-^ in the CNT with a diameter of 0.60 nm in the last extra 400 molecular dynamics simulations.


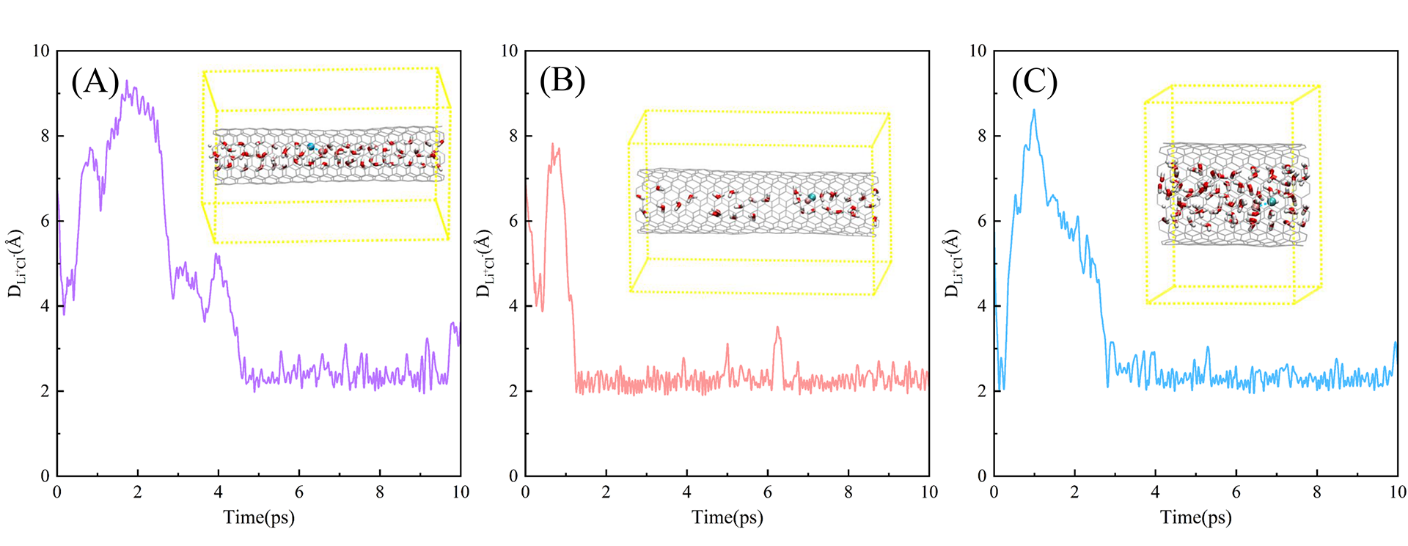


**Supplementary Figure 3.** The distance between Li^+^ and Cl^-^ as a function of time in the CNT with diameters of 0.60 nm (A), 0.73 nm (B), 1.28 nm (C) simulated by first-principles molecular dynamics (AIMD) method.

## Supplementary Tables

**Supplementary Table 1.** The Details of MD simulated systems. Nw is the number of water molecules in CNTs.

| Case | Diameter(nm) | Box size(nm^3^) | N_w_ | ρ_w_(kg/m^3^) |
| --- | --- | --- | --- | --- |
| 1 | 0.60 | 8*8*24.49 | 150 | 0.66 |
| 2 | 0.73 | 8*8*27.19 | 326 | 0.87 |
| 3 | 0.87 | 8*8*20.00 | 326 | 0.83 |
| 4 | 1.00 | 8*8*14.20 | 326 | 0.88 |
| 5 | 1.28 | 8*8*14.20 | 556 | 0.91 |

**Supplementary Table 2.** The Lennard-Jones parameters and partial charges for ions, water molecule (SPC/E), and carbon atom of CNT.

|  | site | σ(nm) | Ε(kJ^.^mol^-1^) | q(e) |
| --- | --- | --- | --- | --- |
| Ions | Li^+^ | 0.213 | 0.076 | 1.000 |
|  | Mg^2+^ | 0.164 | 3.661 | 2.000 |
|  | Cl^-^ | 0.0442 | 0.493 | -1.000 |
| Water | O_w_ | 0.317 | 0.650 | -0.8476 |
|  | H_w_ | 0.000 | 0.000 | 0.4238 |
| CNT | C | 0.355 | 0.293 | 0.000 |

**Supplementary Table 3.** The Details of AIMD systems.

| Diameter (nm) | 0.60 | 0.73 | 1.28 |
| --- | --- | --- | --- |
| **N_C_** | 476 | 544 | 396 |
| **Nw** | 60 | 31 | 74 |
| **Bond_Li-Cl_** | 6.78 | 6.93 | 6.00 |
| **L_CNT_** | 4.13 | 4.14 | 2.17 |
| **Cell** | A=24.50  B=24.48  C=41.74 | A=25.86  B=25.86  C=41.75 | A=29.86  B=29.84  C=22.04 |

**Supplementary Table 4.** The average interaction energy between CNTs and water molecules during molecular dynamics simulations.

| Diameter (nm) | 0.60 | 0.73 | 0.87 | 1.00 | 1.28 |
| --- | --- | --- | --- | --- | --- |
| **E_CNT-H2O_ (kJ/mol)** | -1988.04 | -3669.07 | -3078.96 | -2567.12 | -3691.31 |

# Supplementary Simulation Method

The calculation of Ab-initio Molecular Dynamics (AIMD) are done on the CP2K program.(Kuhne et al., 2020) We obtain a file that can be used as Multiwfn input file by single-point calculation at B97M-rV/6-31G* level and the convergence threshold of density matrix during SCF is set 5.0E-05, in which the structure obtained by classical molecular dynamics simulation, and then Multiwfn 3.8(Lu and Chen, 2012) is used to perform Noncovalent Interaction (NCI) analysis between CNTs and LiCl solution. The hydration of Li^+^ and Mg^2+^ inside only three single-walled infinite armchair CNTs with diameters of 0.60, 0.73 and 1.28 nm at 298 K was simulated by AIMD with periodic boundary conditions. The details of all the simulation cases are listed in Supplementary Table 2. A production simulation phase conducts in the NVT ensemble using the GFN1-xTB method and Canonical sampling through velocity rescaling (CSVR) thermostat. For each configuration, 10 ps of data-production simulations are conducted and a time step of 1.0 fs is used. The molecular visualization program Visual Molecular Dynamics (VMD)(Humphrey et al., 1996) is used to obtain the snapshots of key configuration and analyze the trajectory.

Humphrey, W., Dalke, A., and Schulten, K. (1996). VMD: Visual molecular dynamics. *Journal of Molecular Graphics & Modelling* 14(1)**,** 33-38. doi: 10.1016/0263-7855(96)00018-5.

Kuhne, T.D., Iannuzzi, M., Del Ben, M., Rybkin, V.V., Seewald, P., Stein, F., et al. (2020). CP2K: An electronic structure and molecular dynamics software package - Quickstep: Efficient and accurate electronic structure calculations. *J Chem Phys* 152(19)**,** 194103. doi: 10.1063/5.0007045.

Lu, T., and Chen, F. (2012). Multiwfn: a multifunctional wavefunction analyzer. *J Comput Chem* 33(5)**,** 580-592. doi: 10.1002/jcc.22885.
